# Supplementary material for: Late middle Miocene caviomorph rodents from Tarapoto, Peruvian Amazonia
Source: PLoS One. 2021 Nov 3;16(11):e0258455. doi: 10.1371/journal.pone.0258455 (PMC8565788; doi:10.1371/journal.pone.0258455)
Supplement: S1 File — (DOCX) [file pone.0258455.s001.docx]

# S1 File. Exploring the potential species number of *Microscleromys* from TAR-31 and La Venta.

## Microscleromys from TAR-31

The two species of *Microscleromys* from La Venta (Colombia; late middle Miocene; fig. 24.2G-O, p. 395 [1]), *M. paradoxalis* and *M. cribriphilus*, have been separate on the basis of some differences of their occlusal pattern and especially of their size [1,2], ‘*M. paradoxalis* [being] larger and higher-crowned, whereas *M. cribriphilus* is slightly smaller’ as indicated Walton [1]. The specimens from TAR-31 are very close to these two species of *Microscleromys* in terms of size, global shape and occlusal morphology. *Microscleromys* from TAR-31 is similar to *M. paradoxalis* and *M. cribriphilus* in having high-crowned teeth with a tendency of showing oblique loph(-id)s, not forming lamina, and with flexi(-ids) not full of cement. As *Microscleromys* from TAR-31, *M. paradoxalis* and *M. cribriphilus* have p4s with a complete tetralophodont pattern (IGM 250308; fig. 24.2H, p. 395 [1]) or with a more trilophodont pattern (IGM 251020; fig. 24.2M, p. 395 [1]); lower molars with a groove on the metalophulid I close to the protoconid (IGM 250319 and 250303; fig. 24.2G,L, p. 395 [1]), and without second transverse cristid or the latter, if it is present, is limited to a very short neomesolophid (IGM 251020) or metaconid cristid (IGM 250303; fig. 24.2K, p. 395 [1]); upper teeth with a metaloph reduced or absent (IGM 250321, 251040, 250283, 250320; fig. 24.2I,J,N,O, p. 395 [1]), a third transverse crest absent (IGM 50320) or limited to a short mesoloph lingually free (IGM 250321) or backwardly connected to the posteroloph (IGM 250283) [1]. All these common characters support the attribution of the specimens from TAR-31 to the genus *Microscleromys*.

## Statistical analyses on the material of *Microscleromys*

As the size is an important criterion to distinct the species of *Microscleromys*, we performed statistical analyses on dental measures in order to identify the potential number of species and what species of this genus are present in TAR-31 and La Venta. Three types of statistical analyses were realised on the dental maximum mesiodistal length and linguolabial width by locus (i.e., dp4, p4, m1–2(?), m3(?), dP4, P4, M1–2(?), M3(?)). All these analyzes were performed under R environment v.4.0.3. [3]. For each locus, we made a file including the data and corresponding script(s) (S2 File). As the analyses of m1–2(?) are the most complex, we detailed the corresponding script and used it as a comparative basis for other scripts, which have been adapted for each locus (S2 File).

Firstly, we explored whether the taxonomic groups mentioned by Walton [2] (*M. paradoxalis* nom. nud., *M. cribriphilus* nom. nud., *M.* ?*paradoxalis*, and *Microscleromys* sp.) and our material of *Microscleromys* from TAR-31 are different among them and thus, if they could be consider as different species (H0_null hypothesis_ = taxa of *Microscleromys* from both areas are the same). For this purpose, we performed descriptive statistical (i.e., measurements of central tendencies and dispersion parameters) and visualization (various graphics including boxplots) analyses. *Microscleromys* *paradoxalis* usually corresponds to specimens with the largest tooth length and width, while *Microscleromys* from TAR-31 has medium and the smallest teeth, *M. cribriphilus* occupying an intermediary position or mainly overlapping *M. paradoxalis* or the largest specimens from TAR-31 (Fig S1A). Then, on analysed variables we tested: (i) whether they fulfilled the normality assumptions, in case that some variables did not fulfill these assumptions, we transformed them using log_10_, log_e_ square and inverse functions; (ii) the homoscedasticity of variances with the Bartlett test, this test was made with permutation if the one or both groups are not normal or if it could not be tested; and (iii) the independency and identical distribution of samples with the Bartels and Mann-Kendall tests (‘stats’, ‘RVAideMemoire’ and ‘trend’ packages) [3–5]. As dental length and width may covariate, we tested this with a Pearson correlation test or Spearman correlation test (‘stats’ package). These results showed that there was covariation for all the loci except for dp4s and m3s (see Table S1A). Subsequently, we tested whether there were statistically significant differences among potential taxonomic groups (H0_null hypothesis_= taxa of *Microscleromys* from both places are the same) using the following tests: Kruskal-Wallis, Permutation Analysis of Variance, Student and Wilcoxon. These tests were performed in function of the satisfied previous preconditions. For these analyses we implemented the ‘dunn.test’, ‘RVAideMemoire’, and ‘stats’ packages [3,5,6] (Tables S1B and S1C). Although the dental length and width are closely correlated in almost all cases (Table S1A), they can contain different information (mainly in dp4 and m3(?); Table S1A). Therefore, we tested these statistical differences on the two variables for all loci (Tables S1B and S1C). We obtained disparate results depending on the locus, variable considered and tests realised. The significant differences mostly concern the relation of *Microscleromys* from TAR-31 with *M. paradoxalis* (p4; m1–2(?); M3(?), Kruskal-Wallis test). In these cases, *Microscleromys* from TAR-31 was usually different from the large *M. paradoxalis*, while *M. cribriphilus*-*M. paradoxalis* and *M. cribriphilus-Microscleromys* from TAR-31 were not different. Nevertheless, the results obtained for M1–2(?) showed a significant difference between *Microscleromys* from TAR-31 with *M. cribriphilus* instead of *M. paradoxalis.* In other few cases, *Microscleromys* from TAR-31 was also different from *M. cribriphilus* (length of m1–2(?), Wilcoxon and Kruskal-Wallis tests). These results could be explained by an inappropriate delimitation of the taxonomic groups. As final step of this part, we eliminated the size effect calculating the ratio of the dental length and width and posteriorly performing these tests on it. In the most of cases, the potential taxonomic groups did not show any statistical differences, suggesting that the observed differences among them are mainly explained by the size (Tables S1B and S1C). There are five exceptions to this pattern all concerning *Microscleromys* from TAR-31, two for its relation with *M. paradoxalis* (m1–2(?), Student test; M3(?), Kruskal-Wallis test), one for its relation with *M.* ?*paradoxalis* (P4, Wilcoxon test) and one for its relation with *M.* sp. (p4, Kruskal-Wallis test). It is worth noting that the results obtained should be interpreted with caution because a bias may be present due to a small sample size effect (especially for the material from La Venta) [7,8].


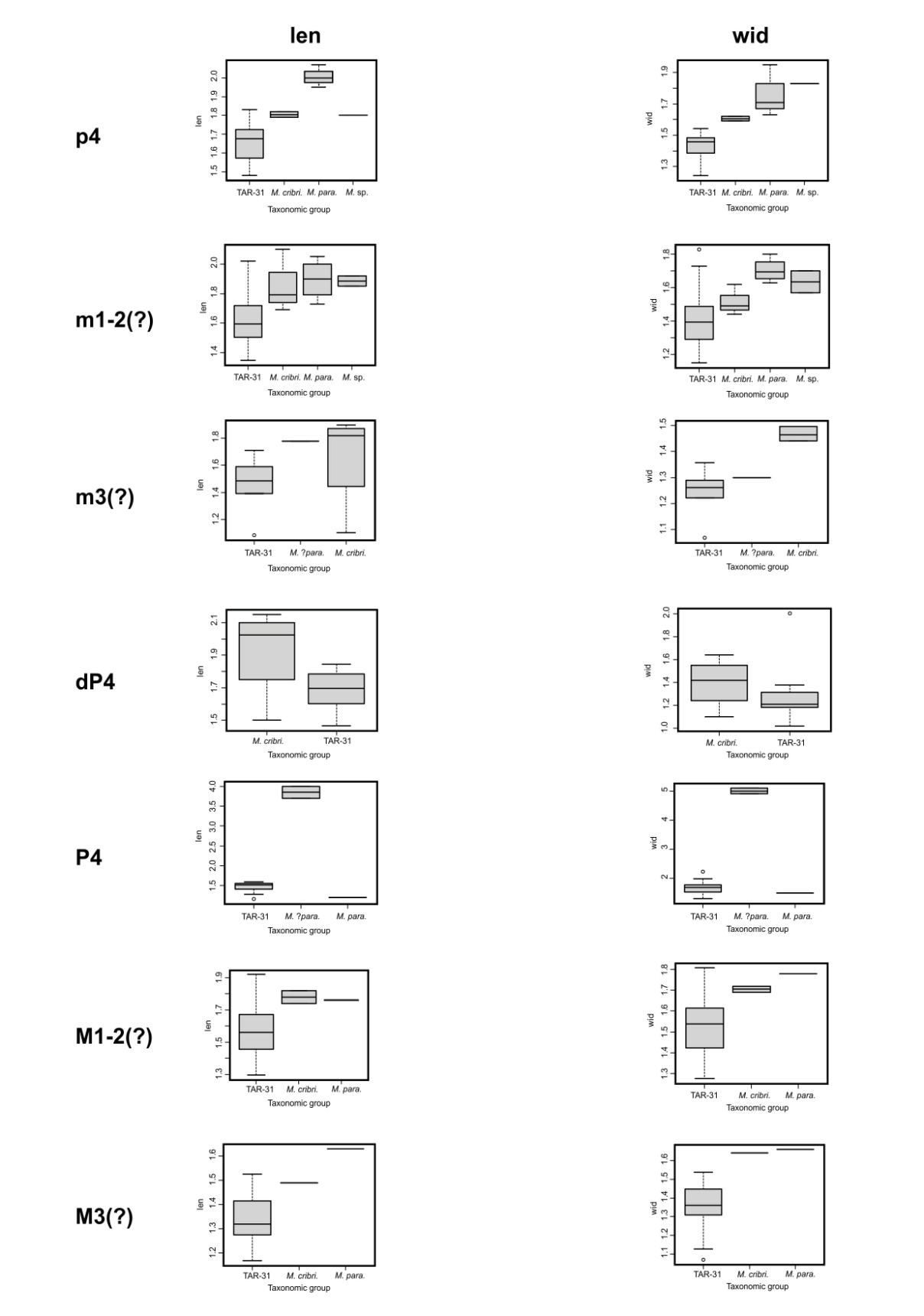


**Fig S1A. Boxplots of the dental length (len) and width (wid) depending on the taxonomic groups for each locus.** *M. cribri*, *M. cribriphilus*; *M. para*, *M. paradoxalis*; *M.* ?*para*, *M.* ?*paradoxalis*; *M.* sp, *Microscleromys* sp.; TAR-31, *Microscleromys* from TAR-31.

Secondly, partitioning analyses were realised until five (dp4), nine (m3) or fifteen (other loci) groups through k-means and k-medoids methods with the ‘cluster’ package [9]. On the graphics of the explained variance from k-means analyses in function of the number of partitions (Figs S1B and S1C), the asymptote is reached for high values: four-five for dp4s (Fig S1B), nine for m3s (Fig S1B) and from nine to 18 for the other loci Figs S1B and S1C). Comparing with the current distribution areas of caviomorph species belonging to a same genus [10], these values are dramatically too high and probably far off the correct value. In addition to the occlusal morphology, the wear can impact the dental size and shape especially in high-crowned species. Thus, these results might be more related to the different degrees of wear between sampling cheek teeth than to a taxonomic signal. We tested this hypothesis for the most numerous loci (i.e., p4, m1–2(?), and M1–2(?)). We reduced our samplings to the teeth showing an intermediate degree of wear. Although reduced due to the elimination of data, the asymptote is still reached for high values (8–9 for p4, 15–16 for m1–2(?), and 12–13 for M1–2(?); results are not showed), which would discard a wear effect. In stratigraphy, fossil specimens from the same geologic bed are assumed that date to the same time period ranging from minutes to months for single-event assemblages or years to tens of thousands of years for attritional assemblages probably such as TAR-31 [11]. This imperfect contemporaneity among specimens of the same bed may constitute another factor, which could hide the taxonomic signal. For all loci and analyses (i.e., total and reduced samplings), the curve slope is maximum for the transition between two and three partitions, numbers more consistent with the biologic data available for these rodents.

**Table S1A. Pearson and Spearman correlation tests between dental lenght and width by locus within *Microscleromys* from La Venta and TAR-31.**

|  | *N* | *Pearson correlation test* | | | *Spearman correlation test* | | |
| --- | --- | --- | --- | --- | --- | --- | --- |
|  |  | *r* | *t* | *P* | *ρ* | *S* | *P* |
|  |  |  |  |  |  |  |  |
| dp4 | 7 | 0.464 | 1.173 | 0.294 |  |  |  |
| p4 | 17 | 0.792 | 5.025 | **1.509e-4** | |  |  |
| m1-2(?) | 68 | 0.831 | 12.133 | **< 2.2e-16** | |  |  |
| m3(?) | 11 | 0.460 | 1.553 | 0.155 |  |  |  |
| dP4 | 21 |  |  |  | 0.625 | 578.000 | **0.003** |
| P4 | 21 |  |  |  | 0.795 | 316.000 | **2.17e-05** |
| M1-2(?) | 44 | 0.654 | 5.605 | **1.467e-06** | |  |  |
| M3(?) | 19 | 0.605 | 3.129 | **0.006** |  |  |  |

Values that are statistically significant (P ≤ 0.05) are in boldface.

*N*, number of specimens; *P*, *P*-value; *r*, r coefficient; *ρ*, rho coefficient; *S*, *S*-statistic; *t*, *t*-statistic.


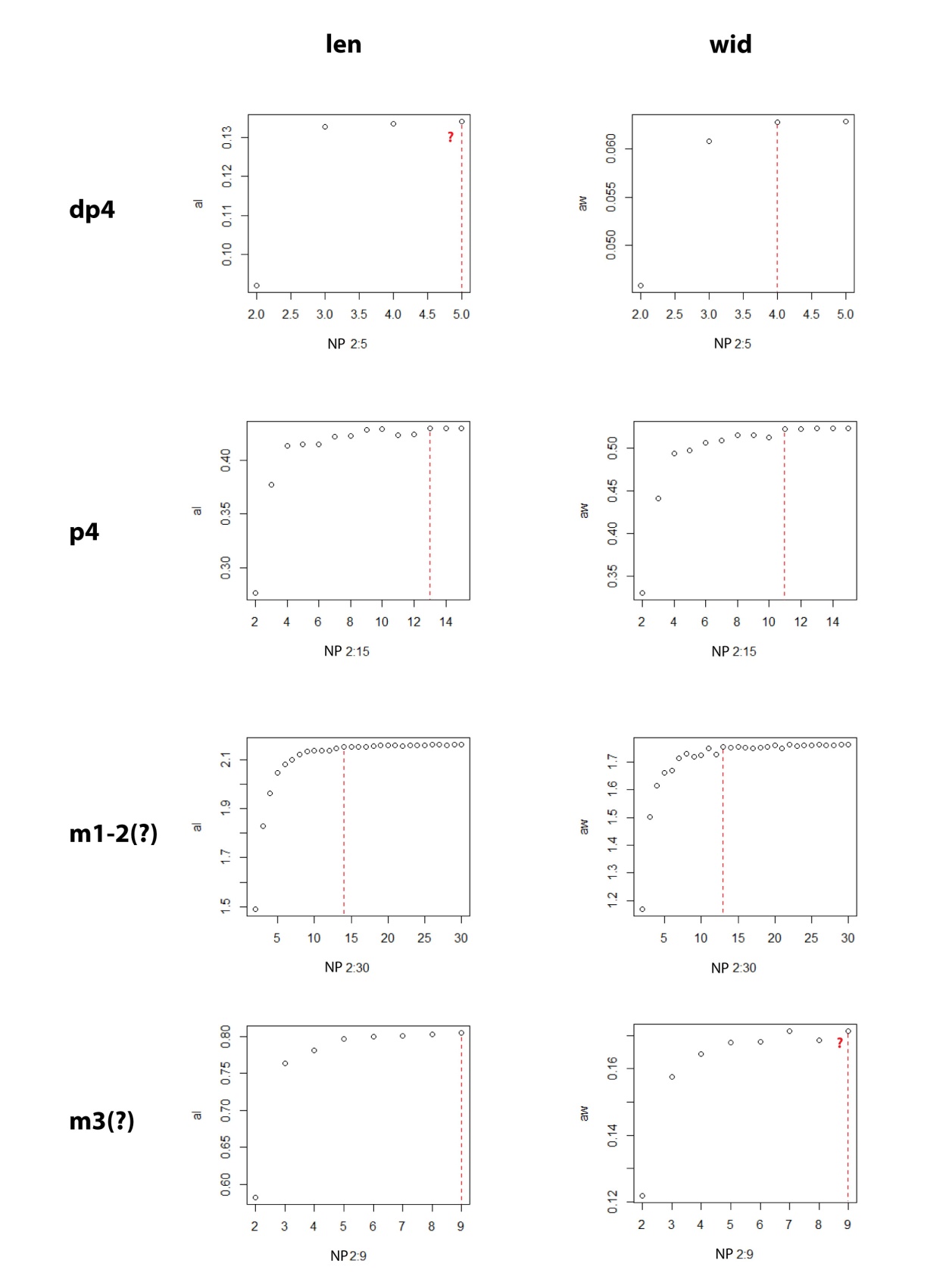


**Fig S1B. Graphics of the explained variance of k-means analyses (al/aw) depending on the number of partitions (NP) for lower teeth.** The dotted line indicates the number of partition for which the asymptote is reached. The question mark is used in the case of uncertainty that the asymptote was reaching. len, length; wid, width.

.
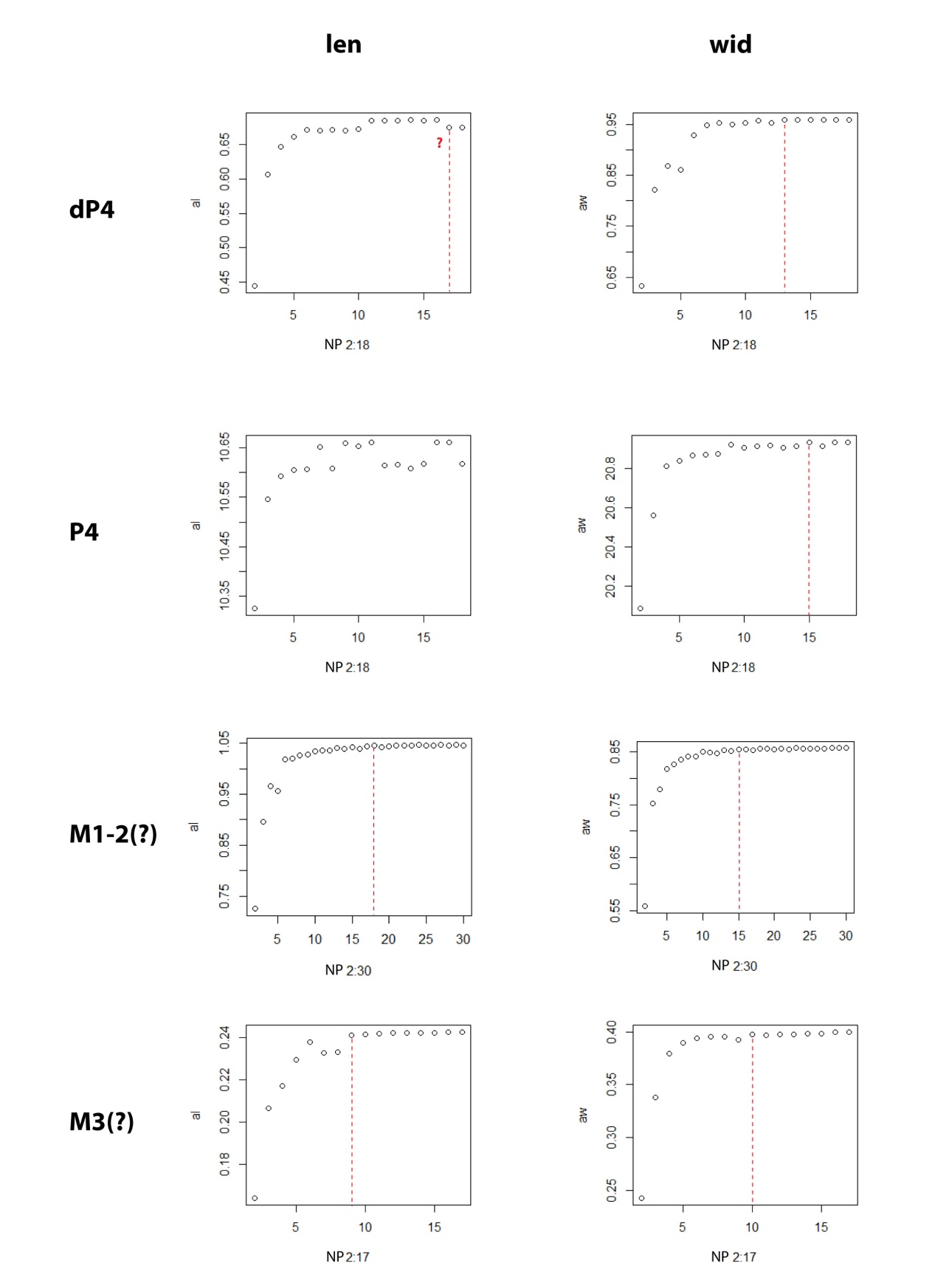


**Fig S1C. Graphics of the explained variance of k-means analyses (al/aw) depending on the number of partitions (NP) for upper teeth.** The dotted line indicates the number of partition for which the asymptote is reached. The question mark is used in the case of uncertainty that the asymptote was reaching. len, length; wid, width.

**Table S1B. Mean comparison tests between the locality and taxonomic groups for the lower teeth.**

|  |  |  | *Wilcoxon test* | | *Student t-test* | | *Kruskal-Wallis test* | | *Permutation Analysis of Variance* | |
| --- | --- | --- | --- | --- | --- | --- | --- | --- | --- | --- |
|  |  |  | *W* | *P* | *t* | *P* | *Χ2* | *P* | *F* | *P* |
| p4 | len | loc: |  |  | 4.391 | **5.259e-4** |  |  |  |  |
|  |  | taxo: |  |  |  |  | 10.032 | **0.0183** | 15.471 | **0.001** |
|  |  | para-cribri: |  |  | 4.356 | 0.188 |  | 0.193 |  | 0.210 |
|  |  | para-TAR31: |  |  | 7.345 | **2.858e-4** |  | **1.600e-3** |  | **0.018** |
|  |  | cribri-TAR31: |  |  | 1.873 | 0.076 |  | 0.070 |  | 0.141 |
|  |  | sp-para: | 3.0 | 0.500 |  |  |  | 0.246 |  |  |
|  |  | sp-cribri: | 1.0 | 1.000 |  |  |  | 0.500 |  |  |
|  |  | sp-TAR31: | 10.0 | 0.333 |  |  |  | 0.139 |  |  |
|  | wid | loc: |  |  | 5.059 | **1.413e-4** |  |  |  |  |
|  |  | taxo: |  |  |  |  | 11.484 | **9.378e-3** | 12.207 | **0.002** |
|  |  | para-cribri: |  |  | 1.270 | 0.184 |  | 0.269 |  | 0.190 |
|  |  | para-TAR31: |  |  | 3.319 | 0.063 |  | **2.300e-3** |  | **0.018** |
|  |  | cribri-TAR31: |  |  | 2.463 | **0.034** |  | **0.047** |  | **0.039** |
|  |  | sp-para: | 1.0 | 1.000 |  |  |  | 0.455 |  |  |
|  |  | sp-cribri: | 2.0 | 0.667 |  |  |  | 0.286 |  |  |
|  |  | sp-TAR31: | 11.0 | 0.167 |  |  |  | **0.029** |  |  |
|  | ratio | loc: |  |  | -1.260 | 0.227 |  |  |  |  |
|  |  | taxo: |  |  |  |  | 3.526 | 0.317 | 0.224 | 0.806 |
|  |  | para-cribri: |  |  | 0.320 | 0.786 |  | 0.269 |  | 0.81 |
|  |  | para-TAR31: |  |  | -0.376 | 0.729 |  | 0.409 |  | 0.81 |
|  |  | cribri-TAR31: |  |  | -0.612 | 0.496 |  | 0.178 |  | 0.81 |
|  |  | sp-para: | 3.0 | 0.500 |  |  |  | 0.077 |  |  |
|  |  | sp-cribri: | 0.0 | 0.667 |  |  |  | 0.187 |  |  |
|  |  | sp-TAR31: | 0.0 | 0.167 |  |  |  | **0.042** |  |  |
| m1-2(?) | len | loc: |  |  | 4.672 | **1.516e-05** |  |  |  |  |
|  |  | taxo: |  |  |  |  |  |  | 7.092 | **0.001** |
|  |  | para-cribri: |  |  | 0.248 | 0.819 |  |  |  | 1.000 |
|  |  | para-TAR31: |  |  | 3.377 | **1.282e-3** |  |  |  | **0.048** |
|  |  | cribri-TAR31: |  |  | 1.929 | 0.187 |  |  |  | 0.084 |
|  |  | sp-para: |  |  | 0.095 | 0.914 |  |  |  | 1.000 |
|  |  | sp-cribri: |  |  | 0.154 | 0.988 |  |  |  | 1.000 |
|  |  | sp-TAR31: |  |  | 2.342 | **0.034** |  |  |  | 0.084 |
|  | wid | loc: |  |  | 4.528 | **2.560e-05** |  |  |  |  |
|  |  | taxo: |  |  |  |  |  |  | 7.952 | **0.001** |
|  |  | para-cribri: |  |  | 2.923 | **0.048** |  |  |  | 0.112 |
|  |  | para-TAR31: |  |  | 4.172 | **9.733e-05** |  |  |  | **0.012** |
|  |  | cribri-TAR31: |  |  | 2.133 | 0.139 |  |  |  | 0.258 |
|  |  | sp-para: |  |  | 1.049 | 0.406 |  |  |  | 0.394 |
|  |  | sp-cribri: |  |  | 1.400 | 0.432 |  |  |  | 0.394 |
|  |  | sp-TAR31: |  |  | 2.288 | 0.052 |  |  |  | 0.112 |
|  | ratio | loc: |  |  | -0.038 | 0.971 |  |  |  |  |
|  |  | taxo: |  |  |  |  | 3.06 | 0.382 |  |  |
|  |  | para-cribri: |  |  | -1.222 | 0.276 |  | 0.052 |  |  |
|  |  | para-TAR31: |  |  | -6.277 | **3.984e-08** |  | 0.077 |  |  |
|  |  | cribri-TAR31: |  |  | 0.652 | 0.581 |  | 0.199 |  |  |
|  |  | sp-para: |  |  | -0.750 | 0.672 |  | 0.310 |  |  |
|  |  | sp-cribri: |  |  | -0.566 | 0.748 |  | 0.188 |  |  |
|  |  | sp-TAR31: |  |  | -0.188 | 0.776 |  | 0.334 |  |  |
| m3(?) | len | loc: |  |  | -1.366 | 0.210 |  |  |  |  |
|  |  | cribri-TAR31: |  |  | -1.089 | 0.292 |  |  |  |  |
|  |  | ?para-TAR31: | 6.0 | 0.286 |  |  |  |  |  |  |
|  |  | ?para-cribri: | 1.0 | 0.800 |  |  |  |  |  |  |
|  | wid | loc: |  |  | -3.501 | **6.708e-3** |  |  |  |  |
|  |  | cribri-TAR31: |  |  | -4.380 | **2.348e-3** |  |  |  |  |
|  |  | ?para-TAR31: | 5.0 | 0.571 |  |  |  |  |  |  |
|  |  | ?para-cribri: | 0.0 | 0.277 |  |  |  |  |  |  |
|  | ratio | loc: |  |  | -0.114 | 0.918 |  |  |  |  |
|  |  | cribri-TAR31: |  |  | 0.285 | 0.764 |  |  |  |  |
|  |  | ?para-TAR31: | 6.0 | 0.286 |  |  |  |  |  |  |
|  |  | ?para-cribri: | 4.0 | 0.400 |  |  |  |  |  |  |

Values that are statistically significant (*P* ≤ 0.05) are in boldface.

cribri, *M. cribriphilus*; *F*, *F*-statistic; len, length; loc, locality (La Venta vs TAR-31); *P*, *P*-value; para, *M. paradoxalis*; ?para, *M.* ?*paradoxalis*; ratio, ratio of the dental length and width; sp, *Microscleromys* sp.; TAR-31, *Microscleromys* from TAR-31; *t*, *t*-statistic; taxo, taxonomic groups; *W*, *W*-statistic; wid, width; *Χ2,* Chi-statistic.

**Table S1C. Mean comparison tests between the locality and taxonomic groups for the upper teeth.**

|  |  |  | *Wilcoxon test* | | *Student t-test* | | *Kruskal-Wallis test* | |
| --- | --- | --- | --- | --- | --- | --- | --- | --- |
|  |  |  | *W* | *P* | *t* | *P* | *Χ2* | *P* |
| dP4 | len | loc & cribri-TAR31: |  |  | 1.665 | 0.187 |  |  |
|  | wid | loc & cribri-TAR31: |  |  | 1.086 | 0.314 |  |  |
|  | 1/wid | loc & cribri-TAR31: |  |  | -1.238 | 0.231 |  |  |
|  | ratio | loc & cribri-TAR31: |  |  | 0.370 | 0.716 |  |  |
| P4 | len | loc: | 17.0 | 0.356 |  |  |  |  |
|  |  | ?para-TAR31: |  |  | -26.300 | **0.008** |  |  |
|  |  | para-TAR31: | 17.0 | 0.211 |  |  |  |  |
|  |  | ?para-para: | 2.0 | 0.667 |  |  |  |  |
|  | wid | loc: |  |  | 1.844 | 0.206 |  |  |
|  |  | ?para-TAR31: |  |  | -20.939 | **0.020** |  |  |
|  |  | para-TAR31: | 15.0 | 0.421 |  |  |  |  |
|  |  | ?para-para: | 2.0 | 0.667 |  |  |  |  |
|  | ratio | loc: |  |  | -4.880 | **4.552e-4** |  |  |
|  |  | ?para-TAR31: |  |  | 2.167 | 0.082 |  |  |
|  |  | para-TAR31: | 16.0 | 0.316 |  |  |  |  |
|  |  | ?para-para: | 0.0 | 0.667 |  |  |  |  |
| M1-2(?) | len | loc: |  |  | 2.417 | **0.020** |  |  |
|  |  | taxo: |  |  |  |  | 5.976 | **0.050** |
|  |  | para-cribri: | 1.0 | 1.000 |  |  |  | 0.500 |
|  |  | para-TAR31: | 38.0 | 0.161 |  |  |  | 0.074 |
|  |  | cribri-TAR31: |  |  | 2.034 | 0.070 |  | **0.022** |
|  | wid | loc: |  |  | 2.605 | **0.013** |  |  |
|  |  | taxo: |  |  |  |  | 5.800 | 0.055 |
|  |  | para-cribri: | 2.0 | 0.667 |  |  |  | 0.412 |
|  |  | para-TAR31: | 39.0 | 0.138 |  |  |  | 0.055 |
|  |  | cribri-TAR31: |  |  | 1.873 | 0.094 |  | **0.032** |
|  | ratio | loc: |  |  | -0.012 | 0.991 |  |  |
|  |  | taxo: |  |  |  |  | 0.683 | 0.711 |
|  |  | para-cribri: | 0.0 | 0.667 |  |  |  | 0.204 |
|  |  | para-TAR31: | 13.0 | 0.564 |  |  |  | 0.257 |
|  |  | cribri-TAR31: |  |  | 0.295 | 0.736 |  | 0.314 |
| M3(?) | len | loc: | 1.0 | 0.023 |  |  |  |  |
|  |  | taxo: |  |  |  |  | 4.581 | 0.101 |
|  |  | para-cribri: | 1.0 | 1.0 |  |  |  | 0.401 |
|  |  | para-TAR31: | 17.0 | 0.111 |  |  |  | **0.043** |
|  |  | cribri-TAR31: | 16.0 | 0.222 |  |  |  | 0.085 |
|  | wid | loc: | 0.0 | 0.012 |  |  |  |  |
|  |  | taxo: |  |  |  |  | 5.116 | 0.077 |
|  |  | para-cribri: | 1.0 | 1.0 |  |  |  | 0.450 |
|  |  | para-TAR31: | 17.0 | 0.111 |  |  |  | **0.042** |
|  |  | cribri-TAR31: | 17.0 | 0.111 |  |  |  | 0.060 |
|  | ratio | loc: | 21.0 | 0.655 |  |  |  |  |
|  |  | taxo: |  |  |  |  | 1.293 | 0.524 |
|  |  | para-cribri: | 1.0 | 1.0 |  |  |  | 0.1574 |
|  |  | para-TAR31: | 10.0 | 0.889 |  |  |  | 0.3803 |
|  |  | cribri-TAR31: | 3.0 | 0.444 |  |  |  | 0.1408 |

Values that are statistically significant (*P* ≤ 0.05) are in boldface.

cribri, *M. cribriphilus*; len, length; loc, locality (La Venta vs TAR-31); *P*, *P*-value; para, *M. paradoxalis*; ?para, *M.* ?*paradoxalis*; ratio, ratio of the dental length and width; TAR-31, *Microscleromys* from TAR-31; *t*, *t*-statistic; taxo, taxonomic groups; *W*, *W*-statistic; wid, width; *Χ2*, Chi-statistic.

Finally, we performed several generalized linear models of partitioning on the most numerous loci including several specimens previously attributed to *M. cribriphilus* and *M. paradoxalis* with the ‘glm’ function of ‘MASS’ and ‘stats’ packages [3,12]. For our purpose, we considered the “Locality” (i.e., TAR-31 vs La Venta) as a random factor, as this implementation leaves us to randomize a possible locality effect on our data. We tested a total of 141 models: 39 for M1–2(?), 51 for p4 and 51 for m1–2(?). For p4 and m1–2(?), the models tested correspond to:

- Model 1: no difference between the specimens from TAR-31 vs La Venta;
- Model 2: TAR-31 vs La Venta;
- Model 3: TAR-31 vs (*M. paradoxalis* + *M.* sp. from La Venta) vs *M. cribriphilus*;
- Model 4: TAR-31 vs *M. paradoxalis* vs (*M. cribriphilus* + *M.* sp. from La Venta);
- Model 5: TAR-31 vs (*M. paradoxalis* + *M. cribriphilus*) vs *M.* sp. from La Venta;
- Model 6: TAR-31 vs *M. paradoxalis* vs *M. cribriphilus* vs *M.* sp. from La Venta;
- Model 7: species 1 from TAR-31 vs species 2 from TAR-31 vs La Venta;
- Model 8: species 1 from TAR-31 vs species 2 from TAR-31 vs (*M. paradoxalis* + *M.* sp. from La Venta) vs *M. cribriphilus*;
- Model 9: species 1 from TAR-31 vs species 2 from TAR-31 vs *M. paradoxalis* vs (*M. cribriphilus* + *M.* sp. from La Venta);
- Model 10: species 1 from TAR-31 vs species 2 from TAR-31 vs (*M. paradoxalis* + *M. cribriphilus*) vs *M.* sp. from La Venta;
- Model 11: species 1 from TAR-31 vs species 2 from TAR-31 vs *M. paradoxalis* vs *M. cribriphilus* vs *M.* sp. from La Venta;
- Model 12: species 1 from TAR-31 vs (species 2 from TAR-31 + *M. cribriphilus* + *M.* sp. from La Venta) vs *M. paradoxalis*;
- Model 13: species 1 from TAR-31 vs (species 2 from TAR-31 + *M. paradoxalis* + *M.* sp. from La Venta) vs *M. cribriphilus*;
- Model 14: species 1 from TAR-31 vs (species 2 from TAR-31 + *M. paradoxalis* + *M.* *cribriphilus*) vs *M.* sp. from La Venta;
- Model 15: (species 1 from TAR-31 + *M. cribriphilus* + *M.* sp. from La Venta) vs species 2 from TAR-31 vs *M. paradoxalis*;
- Model 16: (species 1 from TAR-31 + *M. paradoxalis* + *M.* sp. from La Venta) vs species 2 from TAR-31 vs *M. cribriphilus*;
- Model 17: (species 1 from TAR-31 + *M. paradoxalis* + *M.* sp. from La Venta) vs species 2 from TAR-31 vs *M.* sp. from La Venta;
- Model 18: (species 1 from TAR-31 + *M. paradoxalis* + *M.* *cribriphilus*) vs (species 2 from TAR-31 + *M. cribriphilus*);
- Model 19: (species 1 from TAR-31 + *M. paradoxalis*) vs (species 2 from TAR-31 + *M. cribriphilus* + *M.* sp. from La Venta);
- Model 20: (species 1 from TAR-31 + *M. cribriphilus* + *M.* sp. from La Venta) vs (species 2 from TAR-31 + *M. paradoxalis*);
- Model 21: (species 1 from TAR-31 + *M. cribriphilus*) vs (species 2 from TAR-31 + *M. paradoxalis* + *M.* sp. from La Venta);
- Model 22: (species 1 from TAR-31 + *M. paradoxalis* + *M. cribriphilus*) vs (species 2 from TAR-31 + *M.* sp. from La Venta);
- Model 23: (species 1 from TAR-31 + *M.* sp. from La Venta) vs (species 2 from TAR-31 + *M. paradoxalis* + *M. cribriphilus*);
- Models 24 to 51: models obtained with the k-medoids method on the dental length and width.

For the models 7 to 23, the species named as 1 and 2 from TAR-31 correspond to a partition of the material from the Peruvian locality into two roughly sized-equivalent groups, a small one (species 1) and a larger one (species 2). For the M1–2(?), the models tested are based on the same models that those for p4 and m1–2(?). They are most simple due to M1 or M2 from La Venta were solely attributed to *M. paradoxalis* or *M. cribriphilus*. We choose the better-fitting models with the Akaike’s criteria; i.e., those with lowest value of the Akaike’s value corrected for small samples (AICc), highest Akaike’s weight (AICwi) and delta AICc (ΔAICc) equal to 0 [13]. For the three loci, the best models correspond to partitions obtained with the k-medoids method for high number of groups (10 for p4; 15 for m1–2(?) and M1–2(?); Tables S1D-F), which match with the previous results of the partitioning analyses (Figs S1B and S1C).

**Table S1D. GLM models of partitioning for p4.**

| len | | | | wid | | | |
| --- | --- | --- | --- | --- | --- | --- | --- |
| Model | AICc | ΔAICc | AICwi | Model | AICc | ΔAICc | AICwi |
|  |  |  |  |  |  |  |  |
| 1 | -10.238 | 83.414 | 6.786E-19 | 1 | -6.892 | 82.272 | 1.034E-18 |
| 2 | -22.291 | 71.361 | 2.811E-16 | 2 | -21.817 | 67.347 | 1.801E-15 |
| 3 | -23.239 | 70.414 | 4.515E-16 | 3 | -23.839 | 65.324 | 4.950E-15 |
| 4 | -27.076 | 66.577 | 3.075E-15 | 4 | -20.756 | 68.407 | 1.060E-15 |
| 5 | -21.529 | 72.124 | 1.920E-16 | 5 | -21.099 | 68.064 | 1.258E-15 |
| 6 | -25.078 | 68.575 | 1.132E-15 | 6 | -22.214 | 66.950 | 2.196E-15 |
| 7 | -29.781 | 63.871 | 1.189E-14 | 7 | -20.508 | 68.656 | 9.359E-16 |
| 8 | -33.323 | 60.329 | 6.989E-14 | 8 | -22.719 | 66.444 | 2.828E-15 |
| 9 | -42.331 | 51.322 | 6.315E-12 | 9 | -19.487 | 69.676 | 5.618E-16 |
| 10 | -30.006 | 63.646 | 1.331E-14 | 10 | -19.846 | 69.318 | 6.721E-16 |
| 11 | -40.337 | 53.316 | 2.330E-12 | 11 | -21.114 | 68.049 | 1.267E-15 |
| 12 | -42.331 | 51.322 | 6.315E-12 | 12 | -19.487 | 69.676 | 5.618E-16 |
| 13 | -33.323 | 60.329 | 6.989E-14 | 13 | -22.719 | 66.444 | 2.828E-15 |
| 14 | -30.006 | 63.646 | 1.331E-14 | 14 | -19.846 | 69.318 | 6.721E-16 |
| 15 | -42.331 | 51.322 | 6.315E-12 | 15 | -19.487 | 69.676 | 5.618E-16 |
| 16 | -33.323 | 60.329 | 6.989E-14 | 16 | -22.719 | 66.444 | 2.828E-15 |
| 17 | -30.006 | 63.646 | 1.331E-14 | 17 | -19.846 | 69.318 | 6.721E-16 |
| 18 | -33.323 | 60.329 | 6.989E-14 | 18 | -22.719 | 66.444 | 2.828E-15 |
| 19 | -42.331 | 51.322 | 6.315E-12 | 19 | -19.487 | 69.676 | 5.618E-16 |
| 20 | -42.331 | 51.322 | 6.315E-12 | 20 | -19.487 | 69.676 | 5.618E-16 |
| 21 | -33.323 | 60.329 | 6.989E-14 | 21 | -22.719 | 66.444 | 2.828E-15 |
| 22 | -30.006 | 63.646 | 1.331E-14 | 22 | -19.846 | 69.318 | 6.721E-16 |
| 23 | -30.006 | 63.646 | 1.331E-14 | 23 | -19.846 | 69.318 | 6.721E-16 |
| 24 | -27.523 | 66.130 | 3.845E-15 | 24 | -21.073 | 68.091 | 1.242E-15 |
| 25 | -21.801 | 71.851 | 2.200E-16 | 25 | -27.493 | 61.671 | 3.076E-14 |
| 26 | -44.586 | 49.067 | 1.950E-11 | 26 | -19.900 | 69.264 | 6.905E-16 |
| 27 | -22.247 | 71.405 | 2.750E-16 | 27 | -42.485 | 46.678 | 5.541E-11 |
| 28 | -56.974 | 36.679 | 9.553E-09 | 28 | -20.583 | 68.581 | 9.715E-16 |
| 29 | -20.795 | 72.858 | 1.330E-16 | 29 | -49.075 | 40.088 | 1.495E-09 |
| 30 | -59.343 | 34.310 | 3.123E-08 | 30 | -18.583 | 70.581 | 3.574E-16 |
| 31 | -23.109 | 70.544 | 4.230E-16 | 31 | -51.599 | 37.565 | 5.279E-09 |
| 32 | -67.566 | 26.087 | 1.906E-06 | 32 | -24.089 | 65.074 | 5.609E-15 |
| 33 | -21.225 | 72.427 | 1.650E-16 | 33 | -55.276 | 33.888 | 3.319E-08 |
| 34 | -75.275 | 18.377 | 9.000E-05 | 34 | -23.567 | 65.596 | 4.321E-15 |
| 35 | -21.568 | 72.085 | 1.958E-16 | 35 | -62.372 | 26.791 | 1.153E-06 |
| 36 | -79.418 | 14.234 | 7.142E-04 | 36 | -22.210 | 66.954 | 2.192E-15 |
| 37 | -19.604 | 74.049 | 7.332E-17 | 37 | -67.956 | 21.207 | 1.881E-05 |
| 38 | -89.643 | 4.010 | 1.186E-01 | 38 | -27.947 | 61.217 | 3.860E-14 |
| 39 | -20.382 | 73.270 | 1.082E-16 | 39 | -80.851 | 8.312 | 1.188E-02 |
| **40** | **-93.653** | **0.000** | **8.806E-01** | 40 | -30.400 | 58.764 | 1.316E-13 |
| 41 | -18.383 | 75.270 | 3.982E-17 | **41** | **-89.163** | **0.000** | **7.579E-01** |
| 42 | -46.852 | 46.801 | 6.054E-11 | 42 | -30.772 | 58.391 | 1.585E-13 |
| 43 | -22.435 | 71.217 | 3.021E-16 | 43 | -76.629 | 12.535 | 1.438E-03 |
| 44 | -39.305 | 54.348 | 1.391E-12 | 44 | -31.924 | 57.239 | 2.820E-13 |
| 45 | -29.070 | 64.582 | 8.336E-15 | 45 | -29.591 | 59.572 | 8.782E-14 |
| 46 | -32.354 | 61.299 | 4.304E-14 | 46 | -24.202 | 64.962 | 5.934E-15 |
| 47 | -21.847 | 71.806 | 2.251E-16 | 47 | -27.111 | 62.053 | 2.541E-14 |
| 48 | -20.825 | 72.828 | 1.350E-16 | 48 | -38.186 | 50.977 | 6.457E-12 |
| 49 | -14.940 | 78.712 | 7.122E-18 | 49 | -53.167 | 35.996 | 1.157E-08 |
| 50 | -35.474 | 58.179 | 2.049E-13 | 50 | -52.832 | 36.331 | 9.780E-09 |
| 51 | -26.168 | 67.485 | 1.953E-15 | 51 | -86.768 | 2.396 | 2.288E-01 |
|  |  |  |  |  |  |  |  |

The best model is in boldface.

AICc, Akaike’s value corrected for small samples; ΔAICc, delta AICc; AICwi, Akaike’s weight; len, length; wid, width.

**Table S1E. GLM models of partitioning for m1–2(?).**

| len | | | | wid | | | |
| --- | --- | --- | --- | --- | --- | --- | --- |
| Model | AICc | ΔAICc | AICwi | Model | AICc | ΔAICc | AICwi |
|  |  |  |  |  |  |  |  |
| 1 | -37.429 | 332.652 | 5.759E-73 | 1 | -51.322 | 365.823 | 3.652E-80 |
| 2 | -54.857 | 315.224 | 3.505E-69 | 2 | -67.715 | 349.431 | 1.325E-76 |
| 3 | -52.941 | 317.140 | 1.345E-69 | 3 | -68.521 | 348.624 | 1.983E-76 |
| 4 | -52.915 | 317.166 | 1.328E-69 | 4 | -67.983 | 349.162 | 1.515E-76 |
| 5 | -52.902 | 317.179 | 1.319E-69 | 5 | -65.897 | 351.248 | 5.339E-77 |
| 6 | -50.946 | 319.135 | 4.961E-70 | 6 | -66.866 | 350.279 | 8.666E-77 |
| 7 | -114.305 | 255.776 | 2.843E-56 | 7 | -103.626 | 313.519 | 8.320E-69 |
| 8 | -112.513 | 257.568 | 1.160E-56 | 8 | -106.606 | 310.540 | 3.691E-68 |
| 9 | -112.449 | 257.632 | 1.124E-56 | 9 | -105.638 | 311.507 | 2.275E-68 |
| 10 | -112.418 | 257.663 | 1.106E-56 | 10 | -101.945 | 315.200 | 3.591E-69 |
| 11 | -110.526 | 259.555 | 4.297E-57 | 11 | -105.228 | 311.917 | 1.854E-68 |
| 12 | -112.449 | 257.632 | 1.124E-56 | 12 | -105.638 | 311.507 | 2.275E-68 |
| 13 | -112.513 | 257.568 | 1.160E-56 | 13 | -106.606 | 310.540 | 3.691E-68 |
| 14 | -112.309 | 257.772 | 1.048E-56 | 14 | -101.642 | 315.504 | 3.085E-69 |
| 15 | -112.449 | 257.632 | 1.124E-56 | 15 | -105.638 | 311.507 | 2.275E-68 |
| 16 | -112.513 | 257.568 | 1.160E-56 | 16 | -106.606 | 310.540 | 3.691E-68 |
| 17 | -112.309 | 257.772 | 1.048E-56 | 17 | -101.642 | 315.504 | 3.085E-69 |
| 18 | -112.513 | 257.568 | 1.160E-56 | 18 | -106.606 | 310.540 | 3.691E-68 |
| 19 | -112.449 | 257.632 | 1.124E-56 | 19 | -105.638 | 311.507 | 2.275E-68 |
| 20 | -112.449 | 257.632 | 1.124E-56 | 20 | -105.638 | 311.507 | 2.275E-68 |
| 21 | -112.513 | 257.568 | 1.160E-56 | 21 | -106.606 | 310.540 | 3.691E-68 |
| 22 | -112.309 | 257.772 | 1.048E-56 | 22 | -101.642 | 315.504 | 3.085E-69 |
| 23 | -112.309 | 257.772 | 1.048E-56 | 23 | -101.642 | 315.504 | 3.085E-69 |
| 24 | -115.632 | 254.449 | 5.519E-56 | 24 | -104.825 | 312.321 | 1.515E-68 |
| 25 | -92.520 | 277.561 | 5.285E-61 | 25 | -124.507 | 292.639 | 2.847E-64 |
| 26 | -163.738 | 206.343 | 1.542E-45 | 26 | -115.967 | 301.179 | 3.980E-66 |
| 27 | -102.906 | 267.175 | 9.514E-59 | 27 | -175.674 | 241.472 | 3.674E-53 |
| 28 | -187.344 | 182.737 | 2.061E-40 | 28 | -122.857 | 294.288 | 1.248E-64 |
| 29 | -107.563 | 262.518 | 9.766E-58 | 29 | -206.536 | 210.610 | 1.848E-46 |
| 30 | -222.146 | 147.935 | 7.433E-33 | 30 | -123.400 | 293.745 | 1.637E-64 |
| 31 | -104.585 | 265.496 | 2.203E-58 | 31 | -227.669 | 189.477 | 7.172E-42 |
| 32 | -244.010 | 126.071 | 4.157E-28 | 32 | -123.290 | 293.856 | 1.549E-64 |
| 33 | -119.976 | 250.105 | 4.844E-55 | 33 | -253.090 | 164.056 | 2.375E-36 |
| 34 | -260.078 | 110.003 | 1.282E-24 | 34 | -128.700 | 288.445 | 2.317E-63 |
| 35 | -119.232 | 250.849 | 3.339E-55 | 35 | -267.154 | 149.991 | 2.690E-33 |
| 36 | -277.575 | 92.506 | 8.081E-21 | 36 | -126.197 | 290.948 | 6.628E-64 |
| 37 | -114.055 | 256.026 | 2.508E-56 | 37 | -297.909 | 119.237 | 1.283E-26 |
| 38 | -304.170 | 65.911 | 4.813E-15 | 38 | -126.615 | 290.530 | 8.168E-64 |
| 39 | -113.468 | 256.613 | 1.871E-56 | 39 | -314.986 | 102.159 | 6.552E-23 |
| 40 | -325.537 | 44.544 | 2.100E-10 | 40 | -123.719 | 293.426 | 1.920E-64 |
| 41 | -110.832 | 259.249 | 5.006E-57 | 41 | -322.870 | 94.275 | 3.376E-21 |
| 42 | -334.095 | 35.986 | 1.515E-08 | 42 | -121.627 | 295.519 | 6.744E-65 |
| 43 | -108.634 | 261.447 | 1.668E-57 | 43 | -342.229 | 74.916 | 5.397E-17 |
| 44 | -346.271 | 23.810 | 6.676E-06 | 44 | -120.833 | 296.312 | 4.536E-65 |
| 45 | -108.101 | 261.979 | 1.278E-57 | 45 | -361.537 | 55.608 | 8.411E-13 |
| 46 | -355.559 | 14.522 | 6.939E-04 | 46 | -122.684 | 294.462 | 1.144E-64 |
| 47 | -115.087 | 254.994 | 4.203E-56 | 47 | -368.703 | 48.442 | 3.026E-11 |
| 48 | -361.139 | 8.942 | 1.130E-02 | 48 | -125.363 | 291.783 | 4.367E-64 |
| 49 | -119.361 | 250.720 | 3.561E-55 | 49 | -395.733 | 21.412 | 2.241E-05 |
| **50** | **-370.081** | **0.000** | **9.880E-01** | 50 | -123.979 | 293.166 | 2.187E-64 |
| 51 | -122.699 | 247.382 | 1.890E-54 | **51** | **-417.145** | **0.000** | **1.000E+00** |
|  |  |  |  |  |  |  |  |

The best model is in boldface.

AICc, Akaike’s value corrected for small samples; ΔAICc, delta AICc; AICwi, highest Akaike’s weight; len, length; wid, width.

**Table S1F. GLM models of partitioning for M1–2(?).**

| len | | | | wid | | | |
| --- | --- | --- | --- | --- | --- | --- | --- |
| Model | AICc | ΔAICc | AICwi | Model | AICc | ΔAICc | AICwi |
|  |  |  |  |  |  |  |  |
| 1 | -35.622 | 225.884 | 7.734E-50 | 1 | -44.438 | 222.442 | 4.340E-49 |
| 2 | -39.353 | 222.154 | 4.994E-49 | 2 | -49.027 | 217.853 | 4.305E-48 |
| 3 | -37.365 | 224.141 | 1.849E-49 | 3 | -47.251 | 219.629 | 1.772E-48 |
| 4 | -86.835 | 174.672 | 1.021E-38 | 4 | -56.056 | 210.824 | 1.447E-46 |
| 5 | -84.875 | 176.632 | 3.832E-39 | 5 | -54.332 | 212.548 | 6.108E-47 |
| 6 | -84.875 | 176.632 | 3.832E-39 | 6 | -54.332 | 212.548 | 6.108E-47 |
| 7 | -84.875 | 176.632 | 3.832E-39 | 7 | -54.332 | 212.548 | 6.108E-47 |
| 8 | -84.875 | 176.632 | 3.832E-39 | 8 | -54.332 | 212.548 | 6.108E-47 |
| 9 | -84.875 | 176.632 | 3.832E-39 | 9 | -54.332 | 212.548 | 6.108E-47 |
| 10 | -84.875 | 176.632 | 3.832E-39 | 10 | -54.332 | 212.548 | 6.108E-47 |
| 11 | -84.875 | 176.632 | 3.832E-39 | 11 | -54.332 | 212.548 | 6.108E-47 |
| 12 | -85.545 | 175.962 | 5.356E-39 | 12 | -57.964 | 208.916 | 3.755E-46 |
| 13 | -51.606 | 209.900 | 2.287E-46 | 13 | -94.900 | 171.980 | 3.937E-38 |
| 14 | -115.086 | 146.421 | 1.392E-32 | 14 | -56.990 | 209.890 | 2.308E-46 |
| 15 | -52.144 | 209.363 | 2.993E-46 | 15 | -131.143 | 135.736 | 2.920E-30 |
| 16 | -132.117 | 129.390 | 6.950E-29 | 16 | -58.710 | 208.170 | 5.452E-46 |
| 17 | -49.812 | 211.695 | 9.324E-47 | 17 | -152.190 | 114.690 | 1.085E-25 |
| 18 | -181.203 | 80.304 | 3.168E-18 | 18 | -56.641 | 210.238 | 1.938E-46 |
| 19 | -55.398 | 206.109 | 1.523E-45 | 19 | -170.596 | 96.284 | 1.077E-21 |
| 20 | -187.156 | 74.351 | 6.215E-17 | 20 | -55.677 | 211.202 | 1.197E-46 |
| 21 | -55.148 | 206.359 | 1.344E-45 | 21 | -183.031 | 83.849 | 5.402E-19 |
| 22 | -199.008 | 62.499 | 2.329E-14 | 22 | -63.805 | 203.074 | 6.968E-45 |
| 23 | -53.168 | 208.339 | 4.994E-46 | 23 | -196.281 | 70.599 | 4.072E-16 |
| 24 | -206.841 | 54.666 | 1.170E-12 | 24 | -64.203 | 202.676 | 8.503E-45 |
| 25 | -52.349 | 209.158 | 3.315E-46 | 25 | -215.004 | 51.876 | 4.736E-12 |
| 26 | -215.610 | 45.896 | 9.382E-11 | 26 | -67.820 | 199.060 | 5.186E-44 |
| 27 | -50.505 | 211.001 | 1.319E-46 | 27 | -224.330 | 42.549 | 5.019E-10 |
| 28 | -233.744 | 27.763 | 8.126E-07 | 28 | -59.029 | 207.851 | 6.395E-46 |
| 29 | -49.856 | 211.650 | 9.534E-47 | 29 | -231.416 | 35.464 | 1.735E-08 |
| 30 | -242.840 | 18.667 | 7.677E-05 | 30 | -61.500 | 205.379 | 2.201E-45 |
| 31 | -48.902 | 212.605 | 5.917E-47 | 31 | -238.727 | 28.153 | 6.712E-07 |
| 32 | -242.294 | 19.213 | 5.842E-05 | 32 | -60.736 | 206.144 | 1.501E-45 |
| 33 | -49.795 | 211.712 | 9.245E-47 | 33 | -254.852 | 12.027 | 2.130E-03 |
| 34 | -249.189 | 12.318 | 1.835E-03 | 34 | -58.777 | 208.102 | 5.640E-46 |
| 35 | -47.901 | 213.606 | 3.587E-47 | 35 | -256.630 | 10.250 | 5.180E-03 |
| 36 | -257.708 | 3.799 | 1.299E-01 | 36 | -61.465 | 205.415 | 2.162E-45 |
| 37 | -46.349 | 215.158 | 1.651E-47 | 37 | -262.940 | 3.940 | 1.215E-01 |
| **38** | **-261.507** | **0.000** | **8.681E-01** | 38 | -64.818 | 202.062 | 1.156E-44 |
| 39 | -44.353 | 217.154 | 6.083E-48 | **39** | **-266.880** | **0.000** | **8.712E-01** |
|  |  |  |  |  |  |  |  |

The best model is in boldface.

AICc, Akaike’s value corrected for small samples; ΔAICc, delta AICc; AICwi, Akaike’s weight; len, length; wid, width.

**Number and delimitation of species of *Microscleromys***

Our results showed that *Microscleromys* from TAR-31 is always smaller in average than *M. paradoxalis* and *M. cribriphilus* except for P4 (Fig S1A). However, the size range of *Microscleromys* from TAR-31 tends to encompass that of *M. cribriphilus* or at least the mean of the distribution of the latter*.* This is particularly true for the dental length. For the two most numerous loci (i.e., m1–2(?) and M1–2(?)), the size ranges of *Microscleromys* from TAR-31 and *M. paradoxalis* overlap. A significant difference between *Microscleromys* from TAR-31 and *M. paradoxali*s is frequently found in the distinct datasets (Tables S1B and S1C). Nevertheless, the heterogeneity (between loci) and inconsistency (significant difference between *M. paradoxalis-Microscleromys* but not for *M. paradoxalis-M. cribriphilus* and *M. cribriphilus-Microscleromys*) of results would suggest that the taxonomic groups are not properly delimited. This assessment is supported by the results obtained for the generalized lineal models on p4, m1–2(?), and M1–2(?). Best models are always among the models based on the results of the partitioning analyses. Nevertheless, as discuss above, the best models correspond to dramatically high number of taxonomic groups, which is not consistent with the biologic data for the extant chinchilloids or caviomorphs [10]. Consequently, we follow as criterion of selection the maximum of curve slope, which suggests a more probable number of groups (three groups), instead of the asymptote in the k-means graphic (Figs S1B and S1C). Our analyses of the occlusal morphology of all the material reveal a great variation for the most numerous loci. Firstly, part of this variation (i.e., changes in outline shape, connection and fusion or not between structures, and opening/closing of flexi(-ids)) can be explained by the wear. Secondly, considering solely teeth at early states of wear, the variations concern more specifically the structures between the metalophulid I and the hypolophid for p4s and m1–2(?)s (i.e., posterior arm of the protoconid, metaconid cristid, mesostylid, and neomesolophid) and between the labial protoloph and posteroloph for M1–2(?)s (i.e., mesostyle, mesoloph, mesolophule, metacone, and metaloph) in terms of presence/absence, development, connection and orientation/obliquity. Particularly, the M1–2(?)s display a very variable mesoloph, which can be either i) connected to a mesolophule, ii) free and transverse, iii) oriented backwardly and connected to the posteroloph (via a spur or not), or iv) absent. For all these configurations of the mesoloph, the metaloph can be present or not. The mesolophule can be present (configuration of mesoloph 1), possibly present (configurations 2 and 3) or absent (configuration 4). Some M1–2(?)s show intermediary patterns between distinct configurations of this crest or combining two configurations. For example, the mesoloph with a configuration 2 has a variable length ranging from medium, short or very short/almost absent, the latter is close to the configuration 4. In addition, MUSM 78, 79, 120 and 122 have a mesoloph connected to a mesolophule and to the posteroloph (configurations 1 and 3). For p4s, m1–2(?)s, and M1–2(?)s, the observed morphologic changes do not match with the three partitions obtaining by the k-medioids method. Considering the latter point as well as the large size range of the loci, it seems to be more parsimonious to recognize only two species of *Microscleromys* mainly based on a size criterion. The delimitation of them was partly based on the results of k-medioids method for two partitions. For convention and simplicity, we prefer keep the names proposed by Walton [1,2], but it is worth noting that (i) the original attribution of some specimens from La Venta has been changed (S1 Table); and (ii) most of morphologic differences between *M. paradoxalis* and *M. cribriphilus* mentioned by Walton [2] are here interpreted as diagnostic characters of the genus or as part of its variation intraspecific. We estimated the size of teeth for each species from the calculation of their area (see Systematic part of the manuscript). According our results, these two species are both present in La Venta and TAR-31, *M. paradoxalis* being more frequently found in La Venta, contrary to *M. cribriphilus* more frequent in TAR-31 (S1 Table). Due to their clearly large size, two P4s from La Venta originally determined as *M.* ?*paradoxalis* (UNC unnumbered specimen and IGM-DU 85-411) are attributed as *Microscleromys* sp. 1 in the present work (S1 Table; see Systematic part of the manuscript).

**References**

**1.** Walton AH. Rodents. In: Kay RF, Madden RH, Cifelli RL, Flynn JJ editors. Vertebrate paleontology in the Neotropics. The Miocene Fauna of La Venta, Colombia. Washington and London: Smithsonian Institution Press; 1997. pp. 392-409.

**2.** Walton AH. Rodents of the La Venta Fauna, Miocene, Colombia: biostratigraphy and paleoenvironmental implications. Ph.D. Thesis, Southern Methodist University. 1990.

**3.** R Core Team. R: a language and environment for statistical computing v.4.0.3. Vienna: R Foundation for Statistical Computing. 2020. Available from: http://www.r-project.org.

**4.** Pohlert T. trend: Non-Parametric Trend Tests and Change-Point Detection. R package version 1.1.4. 2020. Available at: https://CRAN.R-project.org/package=trend.

**5.** Hervé M. RVAideMemoire: Testing and Plotting Procedures for Biostatistics. R package version 0.9-79. 2021. Available from: https://CRAN.R-project.org/package=RVAideMemoire.

**6.** Dinno A. 2017. dunn.test: Dunn's Test of Multiple Comparisons Using Rank Sums. R package version 1.3.5. https://CRAN.R-project.org/package=dunn.test.

**7.** Raudys SJ, Jain AK. Small sample size effects in statistical pattern recognition: Recommendations for practitioners. IEEE Trans. Pattern Anal. Mach. Intell. 1991; 13:252-264.

**8**. Rakotomalala R. Tests de normalité - Techniques empiriques et tests statistiques. Version 2.0. 2008 [cited 2021 July 16]. In: Ricco Rakotomalala Blog [Internet]. Available from: https://eric.univ-lyon2.fr/~ricco/ricco.html.

**9.** Maechler M, Rousseeuw P, Struyf A, Hubert M, Hornik K. cluster: Cluster Analysis Basics and Extensions. R package version 2.1.0. 2019. Available from: https://CRAN.R-project.org/package=cluster.

**10.** Patton JL, Pardiñas UF, d’Elía G. Mammals of South America. Vol. 2: Rodents. 1st ed. Chicago: University of Chicago Press; 2015.

**11.** Behrensmeyer AK, Kidwell SM, Gastaldo RA. Taphonomy and paleobiology. Paleobiology. 2000;26: 103-147.

**12.** Venables WN, Ripley BD. Modern Applied Statistics with S. 4th Edition. New York: Springer; 2002.

**13.** Burnham KP, Anderson DR. Multimodel inference understanding AIC and BIC in model selection. Sociol Methods Res. 2004;33: 261-304.
