# Supplementary material for: Late middle Miocene caviomorph rodents from Tarapoto, Peruvian Amazonia
Source: PLoS One. 2021 Nov 3;16(11):e0258455. doi: 10.1371/journal.pone.0258455 (PMC8565788; doi:10.1371/journal.pone.0258455)
Supplement: Attachment — Submitted filename: Response to Reviewers_3.doc [file pone.0258455.s007.doc]

**JOURNAL Requirements:**

EMail: 08/09/2021

1. *We revised all elements so that our manuscript meets PLOS ONE's style requirements. Compared to PLOS ONE style templates, we noted and changed some minor style mistakes about the Headings 1 and 2, the Figure Citations and the place of the legend of some figures in the text. Moreover, we modified the file names following the indications of the Editor’s mail.*
2. *Yes, we modified in the Acknowledgments:*

*‘We gratefully thank the E. Beucler, O. Bourgeois (LPG, Nantes, France) and the ‘Thème Terre’ of LPG in order to have covered the costs of a journey in Montpellier.’*

*by*

*‘We gratefully thank the E. Beucler and O. Bourgeois (LPG, Nantes, France) in order to have allowed MB realizing a journey in Montpellier.’*

*Moreover, we added the Laboratoire de Planétologie et de Géodynamique de Nantes to the list of our funds.*

*3. We request the permission to Elsevier for using of the Figure 1 in Antoine et al. (2021) and the Figures 1 and 2 in Marivaux et al. (2020). These articles are not licensed under CC BY 4.0. They are Rightslink enabled. So, we replaced the maps (A, B) for new ones and deleted the stratigraphic log (C), as it is was recently published (Marivaux et al. 2020). We cited instead the fig. 2 in Marivaux et al. (2020).*

EMail: 21/09/2021

We've checked your submission and before we can proceed, we need you to address the following issues:

1. Please upload a Response to Reviewers letter which should include a point by point response to each of the points made by the Editor and / or Reviewers. (This should be uploaded as a 'Response to Reviewers' file type.) Please follow this link for more information: http://blogs.PLOS.org/everyone/2011/05/10/how-to-submit-your-revised-manuscript/

*We uploaded correctly as a 'Response to Reviewers' instead of Cover Letter.*

2. Thank you for including your ethics statement on the online submission form:

"No permits were required for the described study, which complied with all relevant regulations. The field on TAR-31 was carried out in the framework of the ongoing cooperation agreement between the ‘Museo de Historia Natural de la Universidad Nacional Mayor San Marcos’ (Lima, Peru) and the ‘Institut des Sciences de l’Evolution de Montpellier-Université de Montpellier’ (France)."

To help ensure that the wording of your manuscript is suitable for publication, would you please also add this statement at the beginning of the Methods section of your manuscript file.

*The sentence “The field work on TAR-31 was carried out in the framework of the ongoing cooperation agreement between the ‘Museo de Historia Natural de la Universidad Nacional Mayor San Marcos’ (Lima, Peru) and the ‘Institut des Sciences de l’Evolution de Montpellier-Université de Montpellier’ (France)." was present in the original manuscript in ‘TAR-31, Tarapoto, Peru’ section of the Material & Methods. We added “No permits were required for the described study, which complied with all relevant regulations.” before.*

3. Please upload a copy of Figures 13, 14, 15 and 16 which you refer to in your text on pages 17, 64, 69 and 82. Or if the figure is no longer to be included as part of the submission please remove all reference to it within the text.

*All these references correspond to figures published in precedent works. Therefore, they are not figures of our manuscript and are not included as part of the submission. In many times in the text, we cited this type of figures in this way: ‘in fig X [reference number of the corresponding work]’ or ‘(fig X [reference number of the corresponding work]’) while the figures of the manuscript are cited as ‘Fig X’. If it is possible, we would like to keep the citation to these figures because they are important for more clarity of our study. In addition, for the systematic section it is important to mention the figuration of the material in the manuscript and in precedent works.*

**REVIEWER 1 comments: pdf file**

Please see Solórzano et al (2020). Late early Miocene caviomorph rodents from Laguna del Laja (~ 37° S), Cura-Mallín Formation, south-central Chile. Journal of South American Earth Sciences, 102, 102658.

*Yes, exactly. Thank you. We changed ‘(Laguna del Laja and Pampa Castillo)’ by ‘(e.g., Laguna del Laja and Pampa Castillo)’. (line 79)*

Despite its much lower diversity the Urumaco rodent fauna (Venezuela) is also interesting...

*Yes, clearly the fauna from Urumaco Formation is very interesting and important in paleontological view. Here we had considered the most diverse caviomorph faunas at low latitudes of South America. As far we know, this fauna includes six caviomorph taxa (Sánchez-Villagra, 2012; Carrillo & Sánchez-Villagra, 2015), while some Peruvian faunas include equal number of taxa or more (e.g., 8 from Fitzcarrald Arch; Tejada-Lara et al. 2015). It is the reason why we preferred to not add it to our list, but we changed the sentence for more clarity: ‘At low latitudes of South America, two remarkable and species-rich local faunas are known, La Venta in Colombia (Honda Group; late middle Miocene) [18] and Acre in Brazil (Solimões Fm. [Formation]; late Miocene; see [19]).’ by ‘At low latitudes of South America, two remarkable and very species-rich caviomorph faunas are known, La Venta in Colombia (Honda Group; late middle Miocene) [18] and Acre in Brazil (Solimões Fm. [Formation]; late Miocene; see [19]).’ (lines 79-82)*

Sorry, but compare with what?

*We delete “and compare”. (line 106)*

I believe this is part of your novel and interesting results. If that is the case, you should be a little more clear in this sentence.

*We changed the sentence by ‘This locality shares taxa or closely related taxa with La Venta fauna described by one of us (AHW) in the framework of her PhD [37]:* Microsteiromys, Ricardomys, *and* Microscleromys.’. *(lines 107-109)*

I am not sure why you stated "Colloncuran". In above paragrahs you considered the studied rodent assemblage from Peru to be Laventan.

*We tentatively assigned to* Nuyuyomys chinqaska *the erethizontoid material from MD-67. According to Antoine et al. (2013, 2016), MD-67 is early middle Miocene (i.e., Colloncuran-Laventan SALMA). To clarify it, we added a sentence in the material and methods: ‘Three isolated teeth from MD-67 locality in Peru (MUSM 1974, 1975, and 4298; early middle Miocene) were originally attributed to cf.* Microsteiromys *sp [31]. They are reassigned here in light of the new material from Tarapoto.’. (lines 238-240)*

but also some doubts abouth the cogeneric afinities betweenn Scleromys spp. in northern and southern South America

*Yes, clearly. We added a sentence with respect to this point: ‘Moreover, some doubts were expressed about the cogeneric afinities between* Scleromys *spp. from northern versus southern South America [18,71].’. (lines 2784-2786). Moreover, we decided to follow the notation “*Scleromys*” proposed by Walton (1997) for our taxon and the other northern species of* Scleromys *and modified consequently the manuscript, Table 2, S1 Table and S2 Table.*

I think this paragraph fits better in the "paleobiogeographical implcations" section

*Yes clearly. We put it in the “paleobiogeographical implcations" section. In this section, we changed the paragraph:*

*‘The moderate or strong faunal similarities between MD-67, Fitzcarrald fauna, CTA-44 Top, TAR-31, and La Venta ([33,34,36] and see this work) suggest that the Eastern Cordillera of the North Andes and Pebas System would have not constituted barriers for mammals dispersals during the middle and earliest late Miocene at least for a region close to the Andes (Western Corridor) [36,44].’*

*by*

*‘With our results, the geographical ranges of* Ricardomys longidens*,* Microscleromys paradoxalis *and* M. cribriphilus *are now extended southerly to Peruvian Amazonia for the Laventan interval. More generally, strong taxonomic affinities between the vertebrate assemblages of La Venta (Colombia) and Fitzcarrald, Madre de Dios (MD-67), Iquitos, Contamana, and TAR-31 in Peruvian Amazonia support the existence of a single “Pebasian” biome throughout the middle–earliest late Miocene, and more specifically during the Laventan SALMA, as shown by their terrestrial and aquatic components (crocodylomorphs, chelonians, marsupials, rodents, primates, notoungulates, astrapotheres, litopterns, sirenians, and/or cetaceans; [21,26,31-34,36,158-160] and see this work). These moderate or strong faunal similarities suggest that the Eastern Cordillera of the North Andes and Pebas System would have not constituted barriers for mammalian dispersals during this interval at least for a region close to the Andes (Western Corridor) [36,44].’ (lines 2869-2880)*

*We changed the order of the concerned citations in the bibliography section and in the manuscript (ex-159 and 160 after ex-177).*

This section appears to contradict with those stated above (also highlighted). With your available data I thing there is not much support for this ideas. Alternatively, you can stated that the idea it is just a plausible scenario that deserve further scrutiny.

*Yes, we agree, we deleted this part and the citations associated in the bibliography section.*

*We thank very much the reviewer 1 for his comments.*

**REVIEWER 2 comments:**

The description and comparison of this material [from La Venta] in the present work is based on published photos and drawings” (lines 195/196). Why did the authors not analyze the actual materials described by Walton? Were they not possible to loan for some reason? Maybe adding an explaining sentence would be advisable.

*Yes, it is very important point. Our original project was that two of our co-authors, Dr. Susan Walton (Springfield Technical Community College, USA) and Myriam Boivin (INECOA, Argentina), meet in USA or France (where the material from Tarapoto was studied before being returned to Peru) with original and/or casts of the material from La Venta and Tarapoto, respectively, in order to study all the material together. However, the covid pandemic and the confinement did not allow to manage it, to make and send casts to each other, or to loan request to the concerned Museums. Therefore, we decided to work from the pictures and drawings from Walton (1990, 1997).*

*We added a sentence in the Material and Methods section (La Venta paragraph) to clarify this point: “For reasons related to the covid pandemic and confinement, the original material from La Venta could not be directly observed.”. (lines 194-195)*

Main comments in the pdf in chronological order:

I think you mixed up the descriptions for (B) and (C)?

Please double check the respective cross references in the text.

*Yes, thank you. We had mixed up in the legend. Moreover, we had mistake in the citation of the holotype of* Microsteiromys jacobsi *using the field number (FMNH PM 54677) instead of the collection number (FMNH PM 54672) in the manuscript. We checked and corrected each citation.*

I suggest to mark the holotype specimen, e.g., with an asterisk symbol

I suggest to use the same symbol in the figures to mark the holotype specimens.

*We changed the mention ‘holotype’ by three asterisks and we added the sentence ‘The asterisks appoint the holotype material.’ in all legends of figures with a holotype.*

*We thank very much the reviewer 2 for his comments.*

**MAIN PERSONNAL MODIFICATIONS:**

*We registered our manuscript and the new taxon names on ZooBank and put the associated links on the manuscript body text.*

*We deeply apologize because we had made three errors in the statistical part in the original version of our manuscript (S1 and S2 Files). Noteworthily, these changes do not have implications for the taxonomic assignment of specimens of* Microscleromys *or for broader systematic conclusions.*

1. *We had calculated the correlation coefficient of Spearman between len and wid for all loci in the first version of our manuscript. However, these variables are normal or not depending on the locus. The correlation coefficient of Spearman is non-parametric and is generally used when two continuous variables have a distribution not normal or if one of them is not normal (Schober et al., 2018). Conversely, the correlation coefficient of Pearson is used in case of two normal continuous variables. Thus, we applied the Pearson coefficient when we had two normal variables. We modified the Table S1A (S1 File) and the scripts (S2 File).*
2. *For the tests of average comparison, we deleted the results of non-parametric tests keeping only those of the parametric tests in case of normal variables. We modified the Table S1B,C (S1 File) and the scripts (S2 File).*
3. *We had made a mistake in the figure names in the S1 File. We modified it. For example, for Table S3A, we put Table S1A.*
